# Exploring the Immunomodulatory Effects of Fasting-Mimicking Diet in Healthy Individuals: A Pilot Study

**DOI:** 10.3390/nu18152514

**Published:** 2026-08-03

**Authors:** Nadia de Gruil, Marij J. P. Welters, Saskia J. Santegoets, Sanne Boekestijn, Sam Kaart, Hanno Pijl, Judith R. Kroep, Sjoerd H. van der Burg

**Affiliations:** 1 Medical Oncology, Leiden University Medical Center, 2333 ZA Leiden, The Netherlands; 2 Experimental Cancer Immunology and Therapy Group, Medical Oncology, Leiden University Medical Center, 2333 ZA Leiden, The Netherlands; 3 Internal Medicine Endocrinology, Leiden University Medical Center, 2333 ZA Leiden, The Netherlands; 4 Oncode Institute, 3521 AL Utrecht, The Netherlands

**Keywords:** fasting-mimicking diet, immune analysis, gene set enrichment analysis, TNFα signaling pathway, transcriptome analysis, healthy volunteers

## Abstract

**Background**: Fasting-mimicking diets (FMDs) induce metabolic changes associated with immunomodulation in preclinical studies, yet little is known about the effects of FMD on the human circulating immune system. **Methods**: In the single-arm FIND pilot study (NCT04833439), six of nine healthy participants completed two cycles of a 4-day FMD separated by 3 weeks of regular diet in a repeated-measures design. Blood samples were collected at baseline, after an overnight fast, and the morning after completion of the second FMD cycle. Peripheral blood mononuclear cells (PBMCs) were analyzed by transcriptomic profiling and spectral flow cytometry. **Results**: FMD was associated with reduced plasma glucose and insulin-like growth factor-1 and increased ketone levels. Transcriptomic analysis showed increased expression of genes involved in the TNFα, IFNγ and IFNα signaling pathway after two FMD cycles. Fifteen of the 39 leading-edge genes of the TNFα signature that have previously been associated with myeloid activity and rapid stress responses corresponded with the increased frequencies of circulating CD14-CD11b-CD11c+ dendritic cells (DCs), CD123+ plasmacytoid DCs, and CD14+ CD16+ intermediate monocytes. Moreover, the frequencies of HLA-DR-expressing CD4+ and CD8+ T-cells increased (*p* < 0.05) after two FMD cycles. **Conclusions**: In healthy volunteers, two cycles of FMD were associated with metabolic changes and alterations in circulating myeloid and T-cell populations consistent with activation of selected inflammatory pathways. These exploratory findings warrant larger validation studies in healthy volunteers in combination with functional analyses.

## 1. Introduction

Short-term fasting (STF) and particularly fasting-mimicking diets (FMDs) exert beneficial effects on disease risk factors and longevity in preclinical models [1]. It may potentiate the treatment of a broad range of chronic diseases, including cancer [1,2,3,4]. STF consists of 2–3 days of fluid-only fasting (beverages, broth, and vegetable/fruit juice up to 500 kcal/day) but has been used to describe other durations and regimens in the past [5]. FMD is a special type of fasting regimen, usually 3–7 days every few weeks or per month, with a maximum of about 1000 kcal per day, providing ≥50% of energy requirement [5]. FMD induces the metabolic effects of fasting without complete food abstinence and nutrient deprivation and is typically plant-based, low in protein and high in plant-based fats [5]. FMDs are still being developed and optimized and may therefore vary with respect to frequency, duration, calorie content, types of food used and palatability.

FMD and STF are investigated clinically as adjuncts to the treatment of a wide range of diseases, such as autoimmune diseases [6,7], neurodegenerative diseases [8,9] and cancer [10,11]. Early clinical studies suggest that FMD-induced metabolic reprogramming reduces immunosuppressive myeloid and regulatory T-cell compartments in peripheral blood, while also enhancing intratumor T helper type 1 (Th1)/cytotoxic responses and enrichment of interferon-gamma (IFNγ) signatures, and is associated with better clinical outcomes in patients with cancer [10,12,13]. A subgroup analysis, comprising a few healthy volunteers, suggested a similar effect on immune cells in the blood [10], although these data are limited. FMD and STF were used to modify cardiometabolic risk factors in the context of disease prevention [14,15,16,17] and in longevity studies on anti-aging processes [18], but little is known about the immunomodulatory effects of metabolic reprogramming by FMD in these healthy participants. This raises the question of how FMD-induced metabolic effects may affect immune cells.

FMD induces metabolic changes by reducing glucose and insulin-like growth factor-1 (IGF-1) levels in the blood. This results in downregulation of the IGF-1 receptor (IGF-1R)–AKT–mTOR pathway, which may subsequently affect immune cell metabolism and differentiation, including T-cell fate decisions [19,20,21,22]. This is particularly relevant as mTOR signaling regulates how T-cells sense and integrate immune signals from antigen-presenting cells such as dendritic cells (DCs), nutrients, and growth- and immunoregulatory factors [23]. Moreover, downregulation of the IGF-1R–AKT–mTOR pathway promotes autophagy, thereby prioritizing internal repair and supporting a process called differential stress resistance (DSR) in healthy cells [1,24]. A sub-study including 15 participants who completed three cycles of a 5-day FMD per month not only showed improved body composition with decreased total body fat, visceral adipose fat and hepatic fat mass, but also revealed an effect on immune cells, namely an increased lymphoid-to-myeloid ratio [14].

The immunomodulatory effects of STF have yet to be thoroughly explored in humans, particularly in the absence of typical confounding factors such as disease or treatment. The immune impacts of FMD outside such contexts and over time have not been systematically studied. To fill this gap, an exploratory pilot study in healthy participants undergoing two 4-day FMD cycles was conducted. Comprehensive immune profiling was performed at both the transcriptomic and protein level using peripheral blood mononuclear cells (PBMCs).

## 2. Materials and Methods

### 2.1. FIND Study

**Figure 1:**
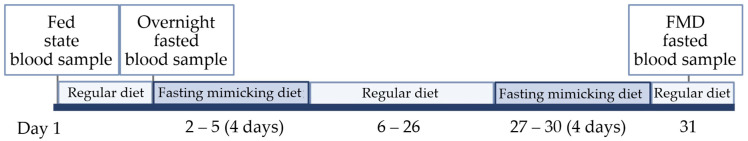
FIND study design. Study design, 2 cycles of a 4-day FMD with a period of about 20 days of regular diet without any restrictions. Blood sampling took place at baseline (fed state), after an overnight fast before the first day of the first FMD cycle (overnight-fasted) and the morning after completion of the second FMD cycle (FMD-fasted). FMD: fasting-mimicking diet.

Healthy volunteers were asked to fast for two cycles using the Xentigen FMD for immune monitoring. Briefly, the main inclusion criteria were: ≥18 years old; body mass index (BMI) ≥ 18.5 and ≤25 kg/m^2^; no chronic diseases, allergies, or use of medication except for contraceptives during the last 12 weeks prior to inclusion. Exclusion criteria were COVID-19 infection or vaccination 6 weeks prior to or during study participation or blood/plasma donation in the last 12 weeks prior to inclusion. Toxicity was investigated at final visit using Common Terminology Criteria for Adverse Events v5.0. The study was conducted in accordance with the Declaration of Helsinki (October 2013, version 1) and approved by the Medical Ethics Committee of Leiden–The Hague–Delft in agreement with Dutch law for medical research involving human subjects and with the General Data Protection Regulation. All healthy volunteers gave written informed consent. Blood was drawn at three time points (Figure 1): at baseline (day 1, fed state), after an overnight fast (day 2, overnight-fasted) and the final blood draw took place the morning after completion of the second FMD cycle (around day 31, FMD-fasted). The FMD Xentigen™ diet (kindly provided by L-Nutra) is for four days, with 1100 kcal on day 1 followed by 240 kcal on days 2–4. Xentigen™ is plant-based, low in calories and amino acids and comprises meals (soups and broths), an energy bar, vitamin and mineral capsule, energy drink, and tea, which the participants were allowed to consume at any time of the designated day. If diet adherence was not possible due to feelings of hunger or lack of energy, an extra 0.5–1 nut bar was allowed on days 2 and 3.

### 2.2. Peripheral Blood Mononuclear Cells (PBMC) Isolation

Venous blood samples (~9 mL) were collected in sodium heparin collection tubes. PBMCs were isolated using Ficoll gradient centrifugation within 6 h of blood withdrawal according to the standard operating procedure at the Medical Oncology Research Laboratory. PBMC samples were cryopreserved in Fetal Calf Serum plus 10% dimethyl sulfoxide and stored in liquid nitrogen for later analysis. Serum was stored at −80 °C before it was analyzed for ketones (β-hydroxybutyrate), IGF-1 and glucose levels in the clinical chemical lab for patient care at the LUMC.

### 2.3. Transcriptomic Analyses

Cryopreserved PBMCs were thawed using the standard operating procedure in medical oncology. RNA was isolated using RNeasy minikit (Qiagen, Venlo, The Netherlands). RNA quality and quantity were assessed using a NanoDrop and Agilent 2100 Bioanalyzer system. Samples had at least 97% of RNA fragments ≥300 nucleotides (>20% required) and corrected RNA concentration was between 82 and 655 ng/μL among samples. The Nanostring nCounter (Bruker Spatial Biology, Seattle, Washington, USA) was performed with the human PanCancer IO360 panel, consisting of 20 housekeeping genes and 750 cancer-associated genes, including immune cell- and immunometabolism-related genes to investigate expression profile changes. Per sample, 200 ng RNA was used as input for the 17 h hybridization with the probes as described previously [25,26]. After quality control in nSolver, the RNA counts were normalized using the nSolver advanced analysis software package (v 1.1.4 ) and according to the guidelines of the software’s developer Bruker. The standard normalization was performed using a combination of normalization on the 6 synthetic ssDNA positive control targets and the housekeeping genes. Linear modeling was used for differentially expressed gene analysis. Benjamini–Hochberg multiple comparison correction was done with the advanced analysis software package of Bruker. Paired analysis was performed in R using the normalized linear and log2 counts.

Gene set enrichment analysis (GSEA) was performed with the freely available Broad Institute GSEA software package (version 4.3.3, www.gsea-msigdb.org, accessed on 27 June 2024) and collections for human gene sets [27], using the nSolver normalized genes from the nCounter dataset. Default setting of 1000 permutations were applied and the human hallmark gene set (h.all.v2023.1.Hs.symbols) was used [28]. Furthermore, default weighted enrichment statistic, default Signal2Noise ranking metric, default max size of 500 and min size of 15, and the Human_Gene_Symbol_with Remapping_MsigDB.v2025.1.Hs.chip were used. Gene sets with a false discovery rate <  0.25 as well as a *p*-value <  0.05 were selected. The FDR threshold of <0.25 was chosen because of the explorative nature of the study, per recommendation by the analysis software package; see: https://docs.gsea-msigdb.org/#GSEA/GSEA_FAQ/#2-why-do-you-recommend-a-false-discovery-rate-fdr-of-025-rather-than-the-more-classic-005-for-gsea (accessed on 27 June 2024).

### 2.4. Multispectral Flow Cytometry

A 40-marker multispectral immunofluorescence panel comprising directly fluorescently labeled antibodies was used for phenotyping myeloid and lymphoid cells in the PBMC samples (three time points per participant), as previously described [29].

### 2.5. Statistical Analysis

The Friedman test and Dunn’s test with adjusted *p*-values were used for post hoc pairwise comparison of metabolic parameters (Dunn–Sidak correction for multiple comparison, two-sided). The Friedman test and Wilcoxon signed rank test post hoc was performed (pairwise and two-sided) for single gene differential expression analysis of the 750 gene nCounter panel, cell type score, pathway signaling score and flow cytometry per subset, using GraphPad Prism 10.2.3 or R 4.2.1 (http://cran.r-project.org, accessed on 8 March 2025). A *p*-value < 0.05 was considered statistically significant.

## 3. Results

### 3.1. FMD Was Tolerable and Safe in Healthy Participants

Sixteen healthy volunteers (participants) were enrolled between November 2021 and February 2022, but seven were ineligible due to COVID-19 infection or COVID-19 vaccination within 4–6 weeks before participation, as depicted in the consort diagram in Supplementary Figure S1. The nine participants who met the inclusion criteria followed a 4-day low-caloric and low-protein FMD cycle twice with a 3-week break without any dietary restrictions. Blood samples were collected at baseline (day 1, fed state), after an overnight fast on the first day of the first FMD cycle (day 2, overnight-fasted state), and on the morning after completion of the second FMD cycle (day 31, FMD-fasted state), as shown in Figure 1.

Nine participants were included for feasibility and toxicity analysis. Six participants completed all measurements, and three participants were missing the final measurement due to interference of COVID-19 infection or vaccination (booster) before final assessment could be conducted. Participant characteristics are shown in Table 1. Six of the nine participants experienced only transient side effects, all probably or possibly related to the FMD, mostly grade 1 (fatigue, muscle weakness). One participant reported a grade-2 headache; no grade-3 or higher side effects were experienced (Supplementary Table S2).

### 3.2. FMD Effectively Modulates Systemic Metabolism from Glycolytic Toward Fatty Acid Oxidation

**Figure 2:**
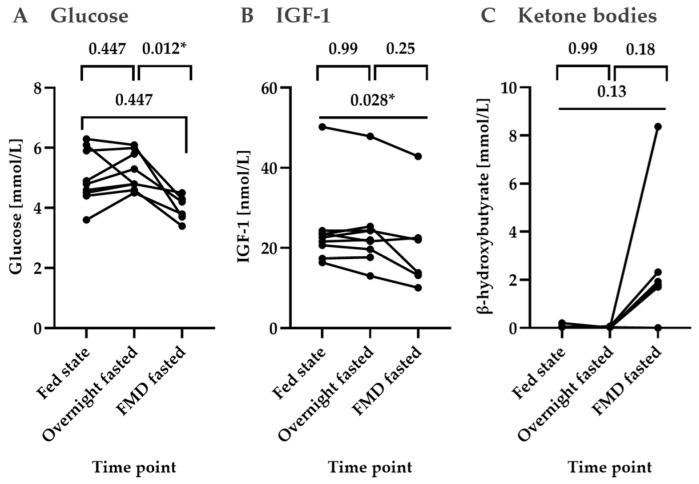
Metabolic markers. Glucose (normal range 3.9–7.7 mmol/L fed; 3.9–5.6 mmol/L fasted), IGF-1 (depending on age within 5.4–47.4 nmol/L for this study group) and ketone levels (normal range 0.02–0.3 mmol/L) were measured and compared at baseline (fed state, day 1), after an overnight fast (overnight-fasted, day 2) and the morning after completion of the second FMD cycle (FMD-fasted, around day 31). Friedman test was used with Dunn’s test for post hoc pairwise comparison, *p*-value adjusted. IGF-1 insulin-like growth factor 1. * level of significance is *p* < 0.05.

To assess the systemic effect of FMD on the body’s metabolism, the plasma levels of glucose, IGF-1 and ketones were measured. The plasma glucose level significantly decreased over time (*p* = 0.012) from the fed state 4.7 mmol/L (median, IQR 4.2–6.0) to FMD-fasted 4.0 mmol/L (median, IQR 3.63–4.35), with the most pronounced difference between overnight-fasted and FMD-fasted (*p* = 0.012; Figure 2). Similarly, IGF-1 plasma levels decreased over time (*p* = 0.029) with the fed state 23 nmol/L (median, IQR 19.6–30.3) and FMD-fasted 17.9 nmol/L (median, IQR 12.4–27.6) showing the largest difference (*p* = 0.028; Figure 2).

Plasma ketone levels, measured as β-hydroxybutyrate, were near zero for all participants at the fed state and overnight-fasted state, but increased at FMD-fasted to a median level of 1.86 mmol/L, with IQR 1.28–3.83, *p* = 0.067 over all three time points and with the largest difference between the fed state and FMD-fasted (*p* = 0.13) as shown in Figure 2. Thus, FMD effectively modulated systemic metabolism from glycolytic toward fatty acid oxidation pathways in these participants.

### 3.3. TNFα, IFNγ, IFNα Pathway Signature Enrichment After FMD

**Figure 3:**
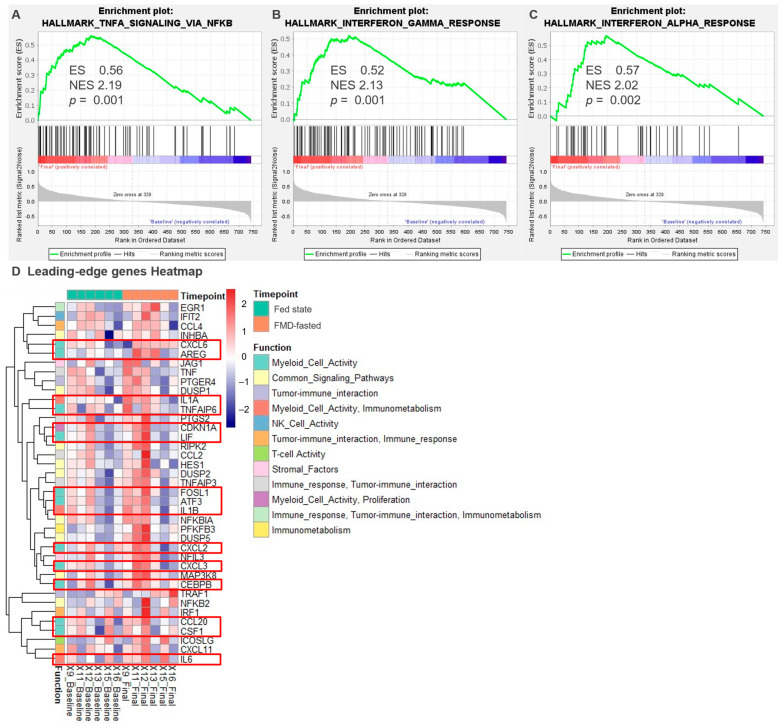
TNFα, IFNγ, IFNα pathway signature enrichment. Gene set enrichment analysis using Nanostring nCounter counts as input, Hallmark gene set to compare, reported gene set with FDR < 25% and *p*-value < 5% and <1%. (**A**) TNFα, (**B**) IFNγ, (**C**) IFNα pathway signature enrichment after two FMD cycles (FMD-fasted state) compared to baseline (fed state), *p* < 0.01 based on 72, 86 and 42 gene expressions respectively. (**D**) Heatmap shows gene expression (log2 fold change) of the 39 leading-edge genes of the TNF gene set with function annotation and the 15 myeloid activity-associated genes outlined in red. NES normalized enrichment score, ES enrichment score, FDR false discovery rate.

To determine FMD-induced changes in circulating immune cells, a transcriptomic analysis on bulk RNA isolated from PBMCs was performed. There was no overt effect on specific genes (Supplementary Figure S2). As it is more likely that the expression of whole sets of genes belonging to specific pathways were altered, rather than a few single genes, GSEA was performed. This revealed an upregulation of genes involved in the tumor necrosis factor alpha (TNFα) pathway, IFNγ pathway and interferon alpha (IFNα) pathway after two FMD cycles (FMD-fasted) compared to the fed state (Figure 3). Fifteen of the 39 leading-edge genes in the TNFα signature via nuclear factor kappa-light-chain-enhancer of activated B cells (NF-κB) are associated with myeloid activity. For example, interleukin 6 promotes macrophage responses in acute innate responses [30,31], and interleukin-1 beta stimulates pro-inflammatory M1 type myeloid activation [32]. In addition, CEPBP (C/EBPβ) and CDKN1A are part of stress-response programs. C/EBPβ is a key transcription factor that induces emergency myelopoiesis and myeloid differentiation [33]. CDKN1A, induced by stress and TNFα transcriptional programs, regulates the cell cycle G1 phase and autophagy, thereby reflecting the cellular stress response [34]. The IFNγ and IFNα leading-edge genes were only strongly upregulated in a few but not all samples (Supplementary Figure S3).

### 3.4. Increased Percentages of Circulating Intermediate Monocytes, Myeloid Dendritic Cells (mDC) and Plasmacytoid DCs (pDC) After Two Cycles of FMD

**Figure 4:**
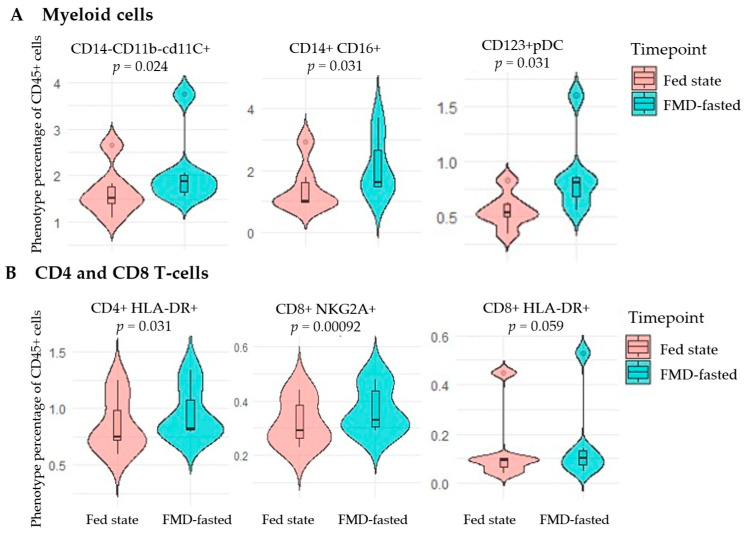
Immunophenotypic changes in percentage of CD45% population. (**A**) Myeloid dendritic cells (CD14-CD11b-CD11c+), intermediate monocytes (CD14+CD16+) and plasmacytoid dendritic cells are increased after two FMD cycles (FMD-fasted) compared to fed state. (**B**) Other cell subsets (CD4+HLA-DR+, CD8+NKG2A+, CD8+HLA-DR+) that are associated with immune activation were increased.

To assess the impact of FMD on the composition and phenotype of circulating immune cells, a flow cytometric analysis was performed. This revealed that the percentages of intermediate monocyte (IM), mDC and pDC subsets were increased after two FMD cycles (FMD-fasted, final) compared to the fed state at baseline (Figure 4A). The increase in these myeloid subpopulations is in line with the enrichment of genes expressed in the TNFα- and IFNα-related transcriptional pathways. IMs are known to mediate inflammatory responses and are responsible for the production of TNF-α and interleukin-1 beta as a rapid response to stressors [35], while CD123+ pDCs are principal producers of type I interferons, and as such may have driven the increased systemic IFNα signature in peripheral blood [36].

### 3.5. An Increase in Activated T-Cells After FMD

The lymphocyte populations were altered after FMD as well. This was reflected by the increases in CD4+HLA-DR+, CD8+NKG2A+, and CD8+HLA-DR+ T-cells in the FMD-fasted state (Figure 4B). Importantly, the expression of HLA-DR and/or NKG2A is likely to reflect the activation of these T-cells [37,38] and may occur after interaction with activated myeloid cells [39].

**Figure 5:**
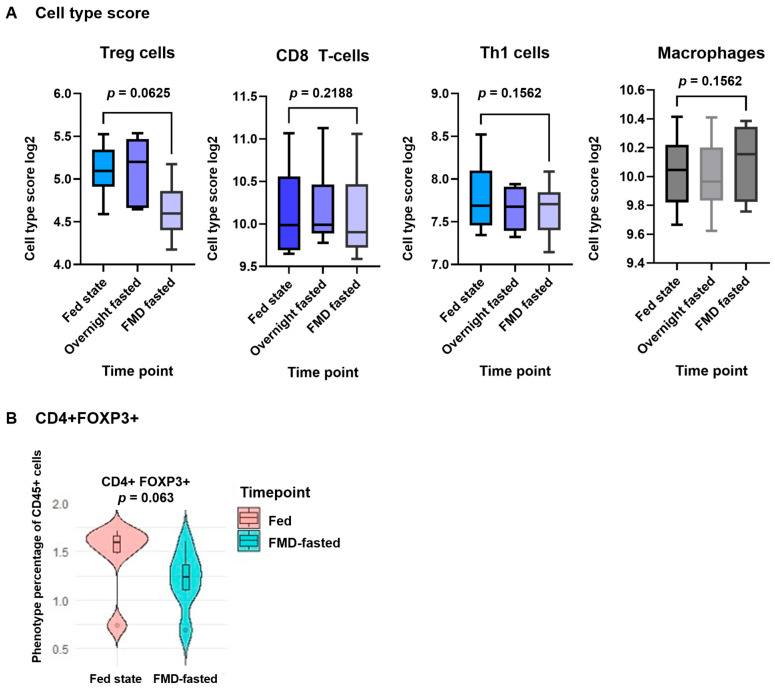
(**A**) Cell type score, which is based on a set of gene expression counts taken together that are characteristically expressed in these immune cell populations and provide an estimate of immune composition in the bulk RNA samples at three time points. (**B**) CD4+FOXP3 Tregs subset as percentage of CD45 showed a decreasing trend at FMD-fasted state compared to fed state. Paired analysis Mann–Whitney U test, 2-sided. Treg, regulatory T-cells; Th1, helper T-cells.

In addition, we observed a trend towards lower percentages of CD4+FOXP3+ regulatory T-cells (Tregs) (*p* = 0.063, Figure 5A) by flow cytometry. This was also observed in our transcriptomic dataset when using the advanced analysis software to estimate cell composition of the bulk RNA sample based on genes that are specific to a cell type. A trend toward Treg downregulation was observed, with a decreasing Treg cell type score in paired analysis between baseline (*p* = 0.0625) and after two FMD cycles, while other major cell type scores (CD8, Th1, macrophages) remained similar (Figure 5B). FMD did not induce changes in circulating lymphoid (CD3+, CD19+, CD56+) to myeloid (CD3-CD19-CD56-) proportions, nor did it remodel the total lymphoid, monocyte (CD14CD16) or dendritic compartments.

## 4. Discussion

This pilot study evaluated the dynamics of the immune system during two cycles of a 4-day FMD in healthy individuals. Our findings showed that short-term FMD may induce marked metabolic and immunological changes. Metabolically, FMD reduced glucose and IGF-1 and elevated ketone levels, reflecting a shift from glycolysis toward fatty acid oxidation. Immunologically, this shift coincided with increased expression of genes involved in the TNFα, IFNγ, and IFNα signaling pathways in PBMCs, and with increased percentages of mDCs, pDCs, IMs, and CD4^+^ and CD8^+^ T-cells expressing activation-associated markers HLA-DR or NKG2A. In addition, a trend toward reduced Tregs was observed. Collectively, these exploratory findings suggest that short-term FMD is associated with changes in cell composition of the immune system consistent with activation of selected inflammatory pathways in healthy individuals.

In contrast to Brandhorst et al., who reported an increased lymphoid-to-myeloid ratio (LMR) following three cycles of a 5-day FMD in an obese, partly prediabetic population [14], we did not observe a shift in the LMR across time points in this cohort. Obesity is known to be associated with baseline expansion of the myeloid compartment, but the FIND cohort included participants with BMI ≤ 25 kg/m^2^. Differences in immune cell assessment methods may also contribute to different results, as LMR in the present study was determined by flow cytometry, whereas Brandhorst et al. determined LMR using a complete blood count [14]. Interestingly, in patients with multiple sclerosis, one cycle of FMD resulted in a decrease in overall lymphocyte numbers [6]. This was not the case in our study, although a trend for a reduction in peripheral Tregs was observed. This is in line with recent reports showing decreased numbers of circulating Tregs [40]. These studies also describe how Treg metabolism relies on glycolysis to maintain suppressive function, activation, and survival through mTOR signaling, whereas FMD is associated with a metabolic shift toward fatty acid oxidation [41]. Although some studies report that upon mTOR inhibition, FOXP3 expression and Treg differentiation increases, such effects may depend on the context, intensity, and duration of nutrient restriction [42]. Alternatively, peripheral reduction may indicate redistribution of Tregs in the body [42,43]. The increased percentage of HLA-DR and/or NKG2A-expressing CD4^+^ and CD8^+^ T-cells indicates T-cell activation after two cycles of FMD [37,38]. It coincided with an increase in fatty acid oxidation, a process required for T memory cells to survive longer and to preserve their capacity to rapidly respond [44]. However, Oudmaijer et al. observed a slightly reduced activation of the central memory subset in healthy living kidney donors after 40h STF [40]. Preclinical studies in tumor-bearing mice consistently demonstrate that FMD or caloric restriction depletes Tregs and enhances CD8^+^ T-cell immunity [12,45].

The increased frequencies of IMs, mDCs and pDCs contrast with earlier work showing a decrease in CD14+ and CD16+ monocytes in healthy volunteers after 19 h of fasting [46]. This difference may be explained by the fact that these cell populations were measured after two cycles of FMD in our current study. Interestingly, Snodgrass et al. reported altered monocyte dynamics after 12 h of overnight fasting and showed that the group with a metabolic profile indicative for lower carbohydrate oxidation but higher fatty oxidation displayed higher numbers of non-canonical CD14dimCD16+ monocytes after fasting [47]. This is in line with our observations of increased IMs coinciding with a shift from glycolytic toward fatty acid oxidation. In addition, our study shows that FMD cycles not only alter monocyte numbers but also increase expression of myeloid-related gene sets, particularly genes involved in the TNFα signaling pathway. This supports a potential role for fasting-induced metabolic stress in shaping innate immune responses. An increased frequency of IMs is also in line with reports indicating that this subset participates in emergency myelopoiesis and rapid immune mobilization during acute stress, such as starvation [40]. The increased percentages of CD11c+ mDCs and CD123+ pDCs may reflect changes in antigen-presenting cell populations, potentially priming the immune system for enhanced responsiveness. This is consistent with the report by Oudmaijer et al., showing the increase in cDC1 (a subset of mDCs) [40]. In line with this, Vernieri et al. observed enhanced IFNγ pathway activation in triple-negative breast cancer patients undergoing repeated FMD cycles [10]. However, intermittent fasting such as Ramadan fasting transiently decreases pro-inflammatory cytokines and circulating immune cells in healthy volunteers, suggesting an overall anti-inflammatory effect [48].

The immune effects of fasting and FMD in humans vary between studies. This is due to differences in setting (healthy, obese, disease, and treatments), types of fasting diet, duration and composition, timing of sampling, metabolic status, and the analytical methods used. Additionally, transient lymphocyte reductions during fasting could precede immune regeneration. Several human studies investigating FMD or STF have not included metabolic validation through circulating ketone measurements [14,40,44,47]. Therefore, it is unclear if the results are from fasting-induced metabolic changes or just caloric restriction. Future clinical studies should focus on healthy participants undergoing FMD, while incorporating metabolic validation and repeated sampling over time.

As this was a pilot study, there are several important limitations, including the small sample size and the lack of a control group. The latter was mitigated by analyzing the data of samples taken before and after FMD intervention over time. As this design did not allow us to fully distinguish FMD-induced changes from individual fluctuations over time or stress, only the most pronounced changes observed in most of the participants were reported here. Another limitation is the inability to link immune composition shifts in IMs, mDCs, pDCs and HLA-DR+ T-cells to specific effector functions or cytokine production. In addition, transcriptomic changes in TNFα, IFNγ, and IFNα pathway signatures may not fully reflect functional outcomes without corroborating data at the protein level or in situ evidence. Moreover, as we analyzed a restricted set of genes focused on immune oncology rather than the whole transcriptome, other changes may have been missed. Functional analyses of monocyte and DC subsets are required to determine whether these FMD-associated immunophenotypic changes are accompanied by altered antigen presentation, cytokine production, or immunosuppressive mechanisms. Furthermore, the impact of the decrease in Tregs should be evaluated functionally.

## 5. Conclusions

In conclusion, our exploratory pilot data supports the hypothesis that FMD is associated with metabolic changes, enrichment of inflammatory pathways, and alterations in circulating innate and adaptive immune cell populations. These preliminary findings require confirmation in larger cohorts with a control group and incorporating metabolic validation (e.g., ketone levels), serial sampling to better understand the kinetics of immune adaptation and functional immune assays to determine whether these phenotypic changes translate into altered immune function.

## Figures and Tables

**Table 1:** FIND participant characteristics.

Patient Characteristic		Value
Age	Median [Years] Range	59 26–62
BMI	median [kg/m^2^]	23.2
	range	20.1–24.4
Sex	male	1
	female	8
Adherence to diet	yes, fully compliant no *	8 1
Toxicity FMD experienced (n)	yes	6
	no	3
Total participants		9

BMI, body mass index; FMD, fasting-mimicking diet. * Minor deviation.

## Data Availability

All study data are presented in the manuscript and Supplementary Materials. The raw data that supports the findings of this study are not available due to data privacy laws; access can be obtained from the corresponding author upon reasonable request.

## Supplementary Materials

The following supporting information can be downloaded at: https://www.mdpi.com/article/10.3390/nu18152514/s1. Supplementary Table S1: Antibody panel spectral flowcytometry for immunophenotyping of PBMC samples; Supplementary Table S2: phenotype overview; Supplementary Table S3: toxicity details; Supplementary Figure S1: FIND CONSORT diagram; Supplementary Figure S2: Volcano plot of Nanostring analysis; Supplementary Figure S3: IFNγ and IFNα heatmaps.

## Abbreviations

The following abbreviations are used in this manuscript:

FMD	Fasting-mimicking diet
STF	Short-term fasting
PBMCs	peripheral blood mononuclear cells
TNFα	tumor necrosis factor alpha
IFNα	Interferon alpha
IFNγ	Interferon gamma
DC	dendritic cells
mDC	myeloid dendritic cells
pDC	plasmacytoid dendritic cells
IM	Intermediate monocytes
Treg	regulatory Tf-cells
Th1	T-helper cell type 1
IGF-1	insulin-like growth factor 1
IGF-1R	insulin-like growth factor 1 receptor
DSR	differential stress resistance
GSEA	gene set enrichment analysis
BMI	body mass index
NF-κB	Nuclear Factor kappa-light-chain-enhancer of activated B cells
LMR	lymphoid-to-myeloid ratio
