# Supplementary material for: Exploring the Immunomodulatory Effects of Fasting-Mimicking Diet in Healthy Individuals: A Pilot Study
Source: Nutrients. 2026 Aug 3;18(15):2514. doi: 10.3390/nu18152514 (PMC13468288; doi:10.3390/nu18152514)

Supplementary material

Supplementary Table S1: Antibody panel spectral flowcytometry for immunophenotyping of PBMC samples.

	Detector	C/BW	Fluor	Antigen	company	Clone	cat number	dilution
UltraViolet channel	UV2	387/15	BUV395	CD45RA	BD	HI100	740298	640
	UV6	443/15	Zombie UV	L/D	biolegend	NA	423107	2400
	UV7	514/28	BUV496	CD16	BD	3G8	612944	80
	UV9	581/31	BUV563	CD39	BD	TU66	4131424	40
	UV10	612/31	BUV615	ICOS	BD	DX29	751092	80
	UV11	664/27	BUV661	CD1c	BD	F10/21A3	750181	40
	UV14	750/30	BUV737	CD86	BD	2331 (FUN-1)	612784	160
	UV16	812/34	BUV805	CD8	BD	SK1	612889	160
violet laser	V1	428/15	BV421	CD161	biolegend	HP-3G10	339914	10
	V2	443/15	SB436	CD123	TFS	6H6	62-1239-42	40
	V3	458/15	PacBlue	CD15	biolegend	W6D3	323022	20
	V4	473/15	BV480	CD33	BD	P67.6	746366	640
	V6	525/17	BV510	CD11c	BD	B-Ly6	563026	40
	V7	542/17	PacOrange	CD3	exbio	UCHT1	PO-514-T100	20
	V8	581/19	BV570	CD45RO	biolegend	UCHL1	304226	40
	V10	615/20	BV605	CD163	biolegend	GHI/61	333616	10
	V11	664/27	BV650	PD1	biolegend	EH12.2H7	329950	40
	V13	720/29	BV711	CD103	biolegend	Ber-ACT8	350222	80
	V14	750/30	BV750	CD56	biolegend	5.1H11	362556	80
	V15	780/30	BV785	CD28	biolegend	CD28.2	302950	80
blue laser	B1	508/20	BB515	CD141	BD	1A4	565084	160
	B2	525/17	AF488	Foxp3	biolegend	259D	320212	40
	B3	542/17	Spark Blue 550	CD14	biolegend	63D6	367148	320
	B8	678/18	PerCP	CD45	biolegend	Hi30	304026	160
	B9	697/19	PerCP/Cy5.5	CD11b	biolegend	ICRF44	301328	80
	B10	717/20	PerCP/eF710	CD274/PD-L1	TFS	MIH1	46-5983-42	20
yellow green laser	YG1	577/20	PE	CLec9a	biolegend	8F9	353804	80
	YG2	598/20	CF568	CD4	antibodies online	C4-206	ABIN6187195	160
	YG3	615/20	PE/Dazzle 594	CD206	biolegend	15-feb	321130	20
	YG4	660/17	PE/Fire640	CD25	biolegend	M-A251	356148	40
	YG5	678/18	PE/Cy5	Tim3	biolegend	F38-2E2	345052	40
	YG7	697/19	PE/Fire700	CD127	biolegend	AO19D5	351366	40
	YG9	780/30	PE/Cy7	KLRG1	biolegend	SA231A2	367720	40
	YG10	812/34	PE/Fire810	HLA-DR	biolegend	L243	307683	160
red laser	R1	660/17	APC	NKG2a	beckman Coulter	Z199	A60797	20
	R2	678/18	Alexa647	CD68	biolegend	Y1/82A	333820	40
	R3	697/19	Spark NIR 685	CD19	biolegend	HIB19	302270	160
	R4	717/20	APC/R700	Lag3	BD	T47-530	565774	80
	R7	783/23	APC/Fire750	CCR7	biolegend	G043H7	353246	20
	R8	812/34	APC/Fire810	CD27	biolegend	QA17A18	B350141	320

Supplementary Table S2: phenotype overview.

Phenotypes		
**CD3-CD19-CD56- myeloid cells**		
HLA-DR+	of Myeloid cells	
CD11b+CD14+		
CD14+CD16+ infl monocytes		of CD14+ cells
CD206- , CD163-		
CD206+ , CD163-		
CD206+ , CD163+		
CD206- , CD163+		
CD14-CD11b-		
CD14-CD11b-/CD11c+		
CD14-CD11b-/CD11c+/CD1c+		
CD1c+/CD163+cDC2		
CD1c+/CD163-cDC2		
CD11c+/cDC1		
CD14-CD11b-/CD11c-		
CD123+ pDC		
HLA-DR-		
DN		
gMDSC		
mMDSC		
**B cells**		
CD27- , CD86-		of B cells
CD27+ , CD86-		
CD27+ , CD86+		
CD27- , CD86+		
**CD56+ NK cells**		
CD16- NK cells		of NK cells
CD56+ NK cells		
**T cells**		
**CD8**	of T cells	
NKG2a		of CD8
CD45RA- , CCR7-		
CD45RA+ , CCR7-		
CD45RA+ , CCR7+		
CD45RA- , CCR7+		
CD45RO- , CD45RA-		
CD45RO+ , CD45RA-		
CD45RO+ , CD45RA+		
CD45RO- , CD45RA+		
CD28- , CD27-		
CD28+ , CD27-		
CD28+ , CD27+		
CD28- , CD27+		
KLRG1+		
HLA-DR+		
ICOS+		
TIM3+		
LAG3+		
PD-1+		
CD103+		
CD39+		
CD161+		
**CD4**	of T cells	
CD25+CD127-FOXP3+ (Treg)		of CD4
CD45RA- , CCR7+		
CD45RA+ , CCR7+		
CD45RA+ , CCR7-		
CD45RA- , CCR7-		
CD28- , CD27+		
CD28+ , CD27+		
CD28+ , CD27-		
CD28- , CD27-		
CD45RO- , CD45RA+		
CD45RO+ , CD45RA+		
CD45RO+ , CD45RA-		
CD45RO- , CD45RA-		
KLRG1+		
CD161+		
CD39+		
CD103+		
PD1+		
LAG3+		
TIM3+		
ICOS+		
HLA-DR+		

Supplementary Table S3: toxicity details

**toxicity symptom**	**nr participants**	**grade**
headache	1	2
dizziness	1	1
weakness	4	1
orthostasis	1	1
feeling cold	1	1
trembling	1	1

Supplementary Figure S1: FIND CONSORT diagram.

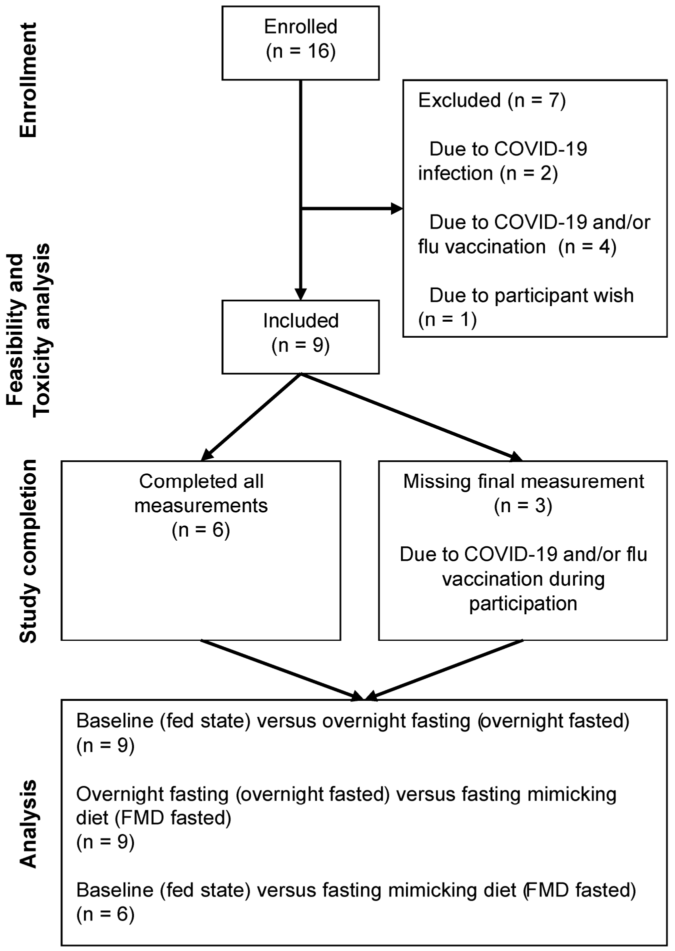

Supplementary Figure S2: Volcano plot of Nanostring analysis.

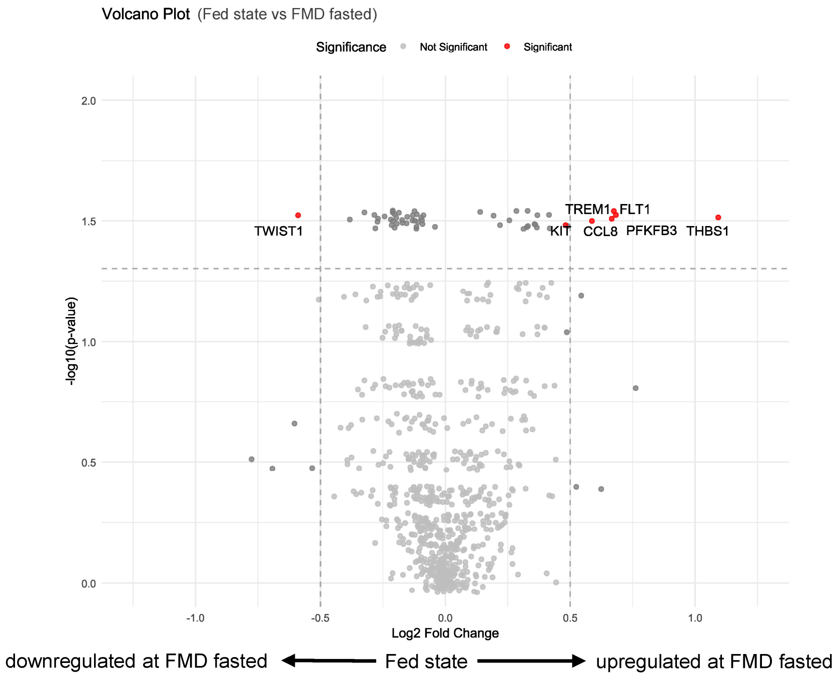

Volcanoplot of log 2-fold change of the 750 gene panel transcriptomic analysis between fed state and FMD fasted. –log10(p-value) is not corrected for multiple testing.

Supplementary Figure S3: IFNγ and IFNα heatmaps.

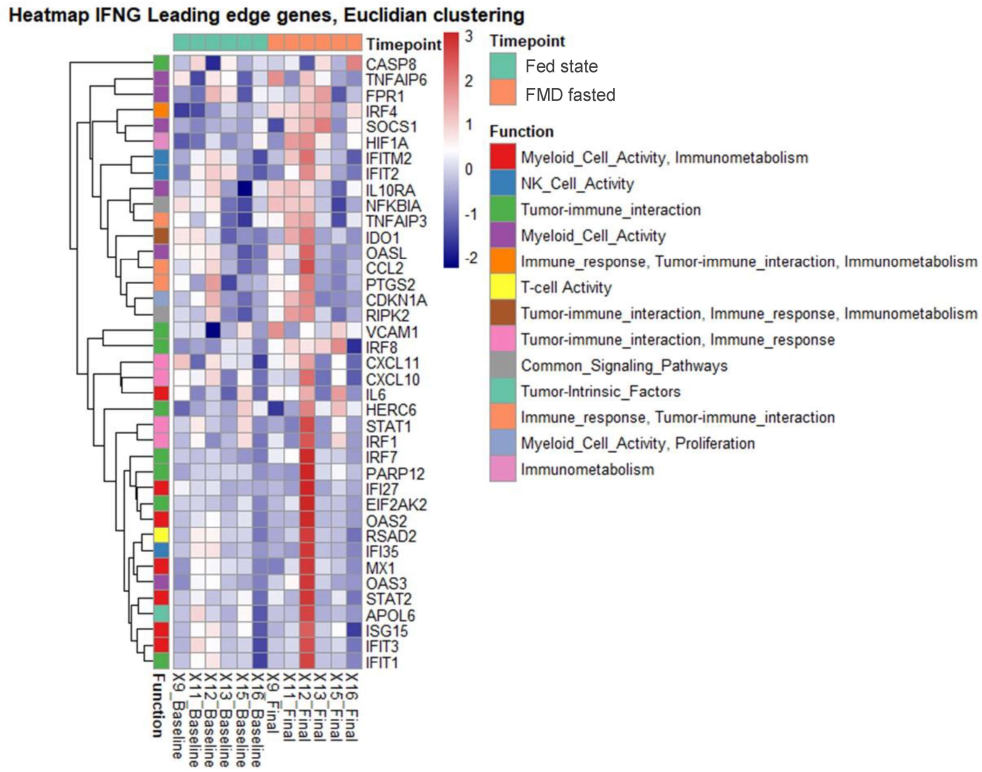

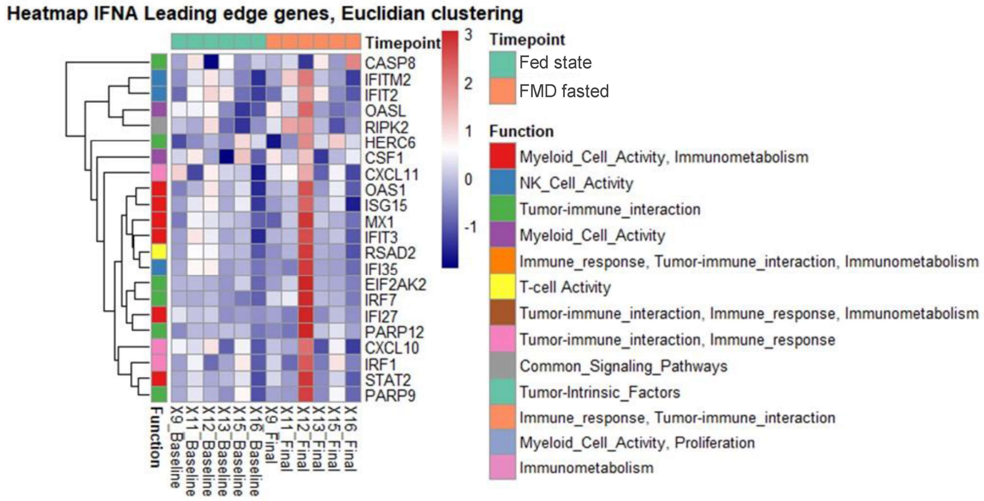

Supplement: Supplementary file 1 [file nutrients-18-02514-s001.zip › nutrients-4391268-supplementary.pdf]
